# Correlation between meteorological factors and vitamin D status under different season

**DOI:** 10.1038/s41598-023-31698-2

**Published:** 2023-03-23

**Authors:** Xichao Wang, Ke Lu, Junjie Shen, Shihan Xu, Qi Wang, Yaqin Gong, Yunyu Xia, Xiaochun Wang, Lin Chen, Shanjun Yan, Zaixiang Tang, Chong Li

**Affiliations:** 1 Department of Biostatistics, School of Public Health, Medical College of Soochow University, Suzhou, 215123 China; 2 Jiangsu Key Laboratory of Preventive and Translational Medicine for Geriatric Diseases, Medical College of Soochow University, Suzhou, 215123 China; 3 Department of Orthopedics, Affiliated Kunshan Hospital of Jiangsu University, No. 91 West of Qianjin Road, Suzhou, 215300 Jiangsu China; 4 Information Department, Affiliated Kunshan Hospital of Jiangsu University, Suzhou, 215300 Jiangsu China; 5 Meteorological Bureau of Kunshan City, Suzhou, 215337 Jiangsu China; 6 Ecology and Environment Bureau of Kunshan City, Suzhou, 215330 Jiangsu China

**Keywords:** Nutrition, Public health

## Abstract

Pregnant women with low vitamin D levels tend to have poor clinical outcomes. Meteorological factors were associated with vitamin D. Here, we aimed to study the current status of 25-Hydroxy vitamin D (25(OH)D) concentrations in pregnant women in Kunshan city and investigate the meteorological factors associated with 25(OH)D levels under different seasons. The correlation between meteorological factors and 25(OH)D levels was estimated by cross-correlation analysis and multivariate logistic regression. A restrictive cubic spline method was used to estimate the non-linear relationship. From 2015 to 2020, a total of 22,090 pregnant women were enrolled in this study. Pregnant women with 25(OH)D concentrations below 50 nmol/l represent 65.85% of the total study population. There is a positive correlation between temperature and 25(OH)D. And there is a protective effect of the higher temperature on vitamin D deficiency. However, in the subgroup analysis, we found that in autumn, high temperatures above 30 °C may lead to a decrease in 25(OH)D levels. This study shows that vitamin D deficiency in pregnant women may widespread in eastern China. There is a potential inverted U-shaped relationship between temperature and 25(OH)D levels, which has implications for understanding of vitamin D changes under different seasons.

## Introduction

Vitamin D is an essential fat-soluble vitamin. 25-Hydroxy vitamin D (25(OH)D) is an important form of vitamin D stored in the human body^1^. The standards for vitamin D levels in the population are still controversial. According to Endocrine Society clinical practice guideline, 25(OH)D concentrations below 30 nmol/l (12 ng/ml) are considered severe vitamin D deficiency. Concentrations below 50 nmol/l (20 ng/ml) are considered to be vitamin D deficiency^2^. Globally, vitamin D deficiency during pregnancy is common, including in China^3–5^. It is estimated that over 90% of women of gestational age have vitamin D levels below 50 nmol/l^4^. Maternal vitamin D status, such as serum 25(OH)D, is essential for pregnancy and infant health outcomes^6–8^. Pregnant women may be at a higher risk of low 25(OH)D levels and related diseases, including preeclampsia^9^, gestational diabetes mellitus^10^, preterm birth^11,12^ and low birthweight^13^. Studies on vitamin D in pregnant women have important public health implications.

Few studies have investigated the prevalence of vitamin D deficiency in pregnant women in eastern China, and most of the available studies have examined the relationship between vitamin D levels and specific diseases. Examples include vitamin D levels and bone disease^14^, vitamin D levels and kidney-related ailments^15,16^. Meteorological factors are crucial to the impact of vitamin D levels. Researchers are increasingly focusing on the relationship between meteorological factors and vitamin D levels. Most previous studies on vitamin D consider the influence of a small number of meteorological factors^17–20^. For example, studies on the prediction of vitamin D deficiency generally ask subjects about their sun exposure habits or the approximate number of hours a week they are exposed to the sun using a questionnaire^21^. In observational studies, researchers generally use some proxy to measure the effect of meteorological factors, such as the number of hours of sunshine per day^22,23^. Whether sunshine hours is a factor affecting vitamin D levels remains a question. The study from southwest China showed a correlation between sunlight hours and vitamin D^22^. However, the survey from Hangzhou, China, gave the opposite result^23^. In recent years, more and more meteorological factors have received the attention of researchers. For example, researchers have looked at the effects of ozone on UV and vitamin D^24^. The relationship between vitamin D status and latitude, cloudiness, UV-B exposure and solar zenith angle was investigated^25–28^. Furthermore, the seasonality of vitamin D has been confirmed in most studies^29–32^. However, to our knowledge, no studies have analyzed the different effects of meteorological factors on vitamin D under different seasons.

Information on the epidemiology of vitamin D status in pregnant women is essential. Exploring the impact of meteorological factors on vitamin D status across seasons is also of great practical importance, particularly in guiding public health policy. However, this information and related studies are limited in the Chinese pregnant women population. The current study collected 25(OH)D data in 22,090 healthy pregnant women in Kunshan, China. The prevalence of vitamin D deficiency was assessed, the correlation between 25(OH)D levels and meteorological factors was explored. We also investigated the non-linear relationships of these factors with serum 25(OH)D in pregnant women under different seasons.

## Methods

### Study population

**Figure 1:**
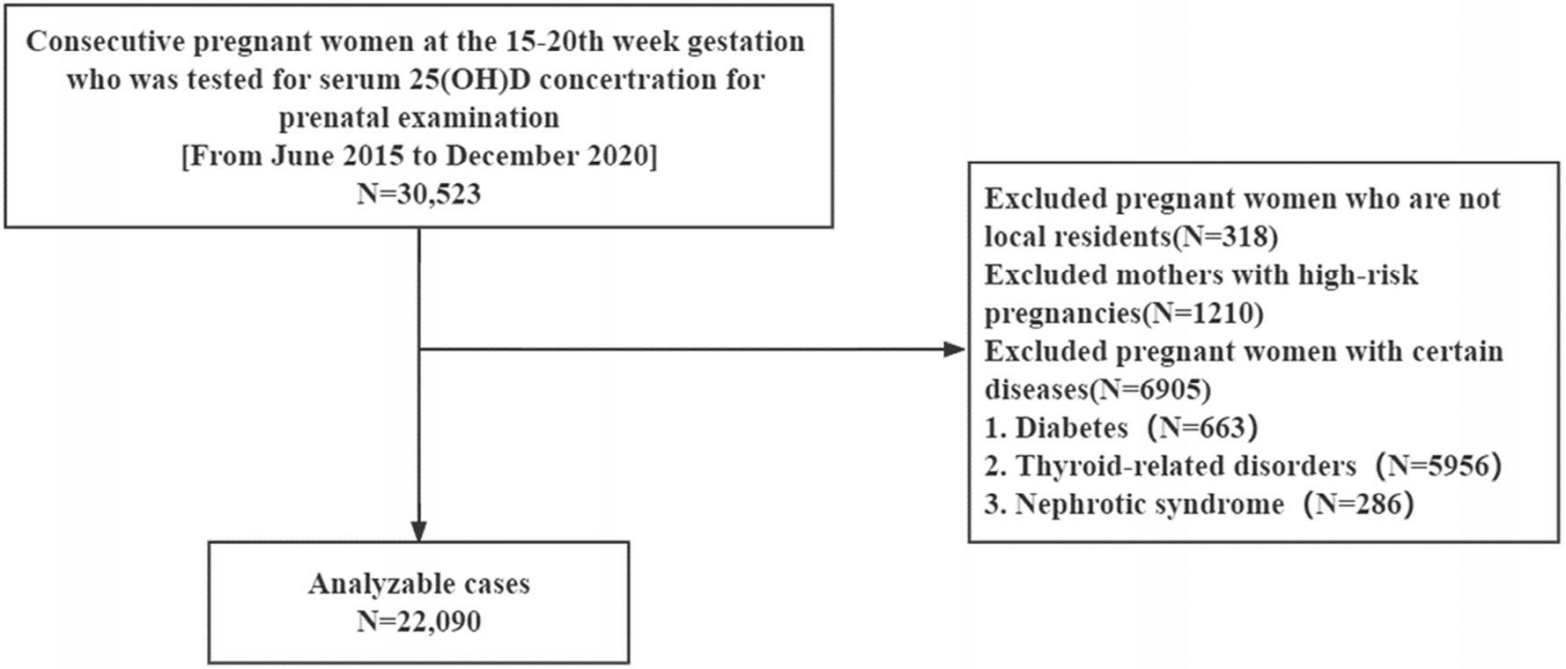
Flow chart for this study.

The data for this study were obtained from Kunshan, China (northern latitude 31°), which has a typical subtropical monsoon climate. From June 2015 to December 2020, 30,523 women in mid-pregnancy (15–20 weeks of gestation) who underwent pregnancy monitoring and serum 25(OH)D concentration testing were collected. As shown in Fig. 1, we excluded pregnant women who are not local residents and excluded those with high-risk pregnancies. (The effect of meteorological factors on vitamin D may require a longer time period. Exclusion of non-local residents would allow the study population to be in a similar meteorological environment as much as possible. Also, we did not collect additional information such as facial skin status of the study population. The inclusion of subjects who were all local residents was also intended to make the study population as consistent as possible.) Pregnant women with certain diseases that with consequences for the metabolism of 25(OH)D were also excluded. A total of 22,090 pregnant women were eventually included in this study. The study protocol, submitted for review by the ethical committee at the Affiliated Kunshan Hospital of Jiangsu University (approval No. 2020-03-046-K01), was approved, and it complied with the Declaration of Helsinki. Patient information was initially documented for hospital’s quality improvement purposes. The requirement for informed consent was waived because of the anonymous and observational design of this investigation, and the decision was approved by the Ethics Committee of Kunshan Hospital, Jiangsu University. Data analyzers were blinded to the identity of the patients.

### Assessment of 25(OH)D

All participants in this study were asked to fast before blood samples were taken. Serum 25(OH)D concentrations were measured immediately using an automated electrochemiluminescence immunoassay on a Roche Cobas 8000/e602 analyzer (Roche Diagnostics, Mannheim, Germany). All 25(OH)D assays in this paper were tested by the same machine and all measurements passed the National Health Council's Endocrine External Quality Assessment.

### Meteorological data

Meteorological data are from the Ecology and Environment Bureau of Kunshan City. Data was matched with patient information. The following meteorological information was collected: daily average temperature, °C; daily average atmospheric pressure, hPa; daily average relative humidity, %; daily average wind speed, m/s (These indicators refer to the arithmetic mean of the observations during one day (24 h).); daily total sunshine hours, hour (The daily total sunshine hours is defined as the sum of the periods of direct solar irradiance at or above 120 w/m^2^.); daily total precipitation, mm (The daily total precipitation represents the depth of precipitation accumulated on a horizontal surface during a day without evaporation, infiltration, or loss).

### Definitions

Based on previous study^2^, for the 25(OH)D levels, we took the following groups: 25(OH)D concentrations below 50 nmol/l are considered to be vitamin D deficiency. Concentrations below 75 nmol/l are considered to be vitamin D insufficient. Concentrations above 75 nmol/l are considered to be sufficient vitamin D levels. (The above groupings are only for data analysis. In the data description section, readers can make their own judgments about vitamin D deficiency based on their preferred criteria.) Seasonal factors in the samples were defined and classified according to spring (March, April, and May), summer (June, July, and August), autumn (September, October, and November), and winter (December, January, and February). We determined age grouping criteria based on several previous studies and surveys^33–36^. Those younger than 25 years old were defined as young maternal, those between 25 and 35 years old were defined as age-appropriate maternal, and those older than 35 years old were defined as advanced maternal.

### Statistical analyses

The baseline characteristics were presented using means with the standard deviations or medians with the interquartile ranges for continuous variables and frequencies with percentages for categorical variables. The Kruskal–Wallis test was used to compare continuous variables, and the chi-square test was used to compare categorical variables. The correlation between meteorological factors and 25(OH)D concentrations was initially estimated by cross-correlation analysis, taking into account the strong correlation between meteorological factors. Univariate and multivariate logistic regression models were fitted separately to explore the relationship between 25(OH)D concentrations and meteorological factors.

To further confirm the consistency between observed meteorological factors and 25(OH)D concentrations, we conducted subgroup analyses according to different seasons (spring, summer, autumn and winter) and different age groups.

To detect any possible non-linear dependence in the regression model and allow flexibility in interpreting the relationship between continuous covariates and study outcomes, changes in meteorological factors were assessed by shape-constrained three-dimensional spline regression models. We fitted shape-constrained cubic spline regression models for the dichotomous dependent variable (presence of vitamin D deficiency) and the continuous dependent variable (25(OH)D levels) in separate waves. Considering our sample size, in order to balance the smoothness of the curve and avoid overfitting, we selected 5 knots. All statistical analyses were performed using R4.1.0 (https://www.r-project.org/). We considered a two-sided *P* value < 0.05 to be significant.

## Results

### Prevalence of serum 25(OH)D deficiency in pregnant women assessed in China

**Figure 2:**
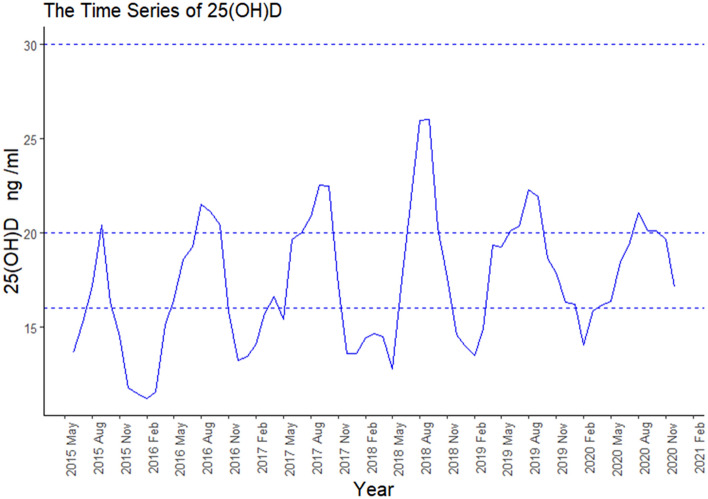
The changing trends of serum concentration of 25(OH)D by year and month.

The median 25(OH)D levels in subjects were 40 nmol/L (IQR 30, 55). From 2015 to 2020, pregnant women with 25(OH)D concentrations below 50 nmol/L represent 65.85% of the total study population. The annual rates were 81.30%, 71.17%, 61.28%, 65.60%, 62.96% and 65.51% respectively. Pregnant women with 25(OH)D concentrations below 75 nmol/L (but above 50 nmol/L) accounted for 25.93% of the total study population. The annual rates were 15.56%, 23.71%, 31.02%, 28.85%, 29.54% and 27.99% respectively. In most cases, 25(OH)D levels in Kunshan pregnant women are low. The mean 25(OH)D levels reached 50 nmol/L (20 ng/ml) only for a few months. As shown in Table 1 and Fig. 2, 25(OH)D levels were significantly higher in summer and autumn than in spring and winter. Although pregnant women's 25(OH)D levels increased each year from 2015 to 2018, this trend became less pronounced from 2018 to 2020.

**Table 1:** Baseline characteristics of participants in the total population under different seasons.

Variable	Spring (n = 5588)	Summer (n = 5905)	Autumn (n = 5435)	Winter (n = 5162)	*P*
Age(years)	29 ± 4	29 ± 4	29 ± 4	29 ± 4	0.14*
25(OH)D (nmol/L)	35(28,48)	48(35,63)	48(35,63)	23(30,43)	< 0.001^^^
< 50 nmol/L	4297 (76.9)	3106 (52.6)	2897 (53.3)	4246 (82.3)	< 0.001^#^
50 ~ 75 nmol/L	1035 (18.5)	2105 (35.6)	1836 (33.8)	752 (14.6)	
> 75 nmol/L	256 (4.6)	694 (11.8)	702 (12.9)	164 (3.2)	
Daily average temperature (°C)	17 ± 5	28 ± 3	19 ± 5	7 ± 4	< 0.001*
Daily total sunshine hours (h)	5.1 ± 4.3	5.1 ± 4.3	4.2 ± 3.7	3.6 ± 3.6	< 0.001*
Daily average pressure (hPa)	1015 ± 6	1005 ± 3	1018 ± 6	1026 ± 5	< 0.001*
Daily average relative humidity (%)	70 ± 16	79 ± 12	78 ± 12	74 ± 14	< 0.001*
Daily total precipitation (mm)	3 ± 7	7 ± 18	4 ± 11	2 ± 5	< 0.001*
Daily average wind speed (m/s)	2.2 ± 0.8	2.1 ± 0.9	1.6 ± 0.7	1.9 ± 0.8	< 0.001*

25(OH)D, 25-hydroxyvitamin D.

^#^*P* was calculated used the chi-squared test.

^^^*P* was calculated used the Kruskal–Wallis test.

*P was calculated used the one-way ANOVA test.

*P* < 0.05 is considered statistically significance.

*P*-values indicate significant differences for the given parameter between the seasons.

### The relationship between meteorological factors and 25(OH)D levels

**Figure 3:**
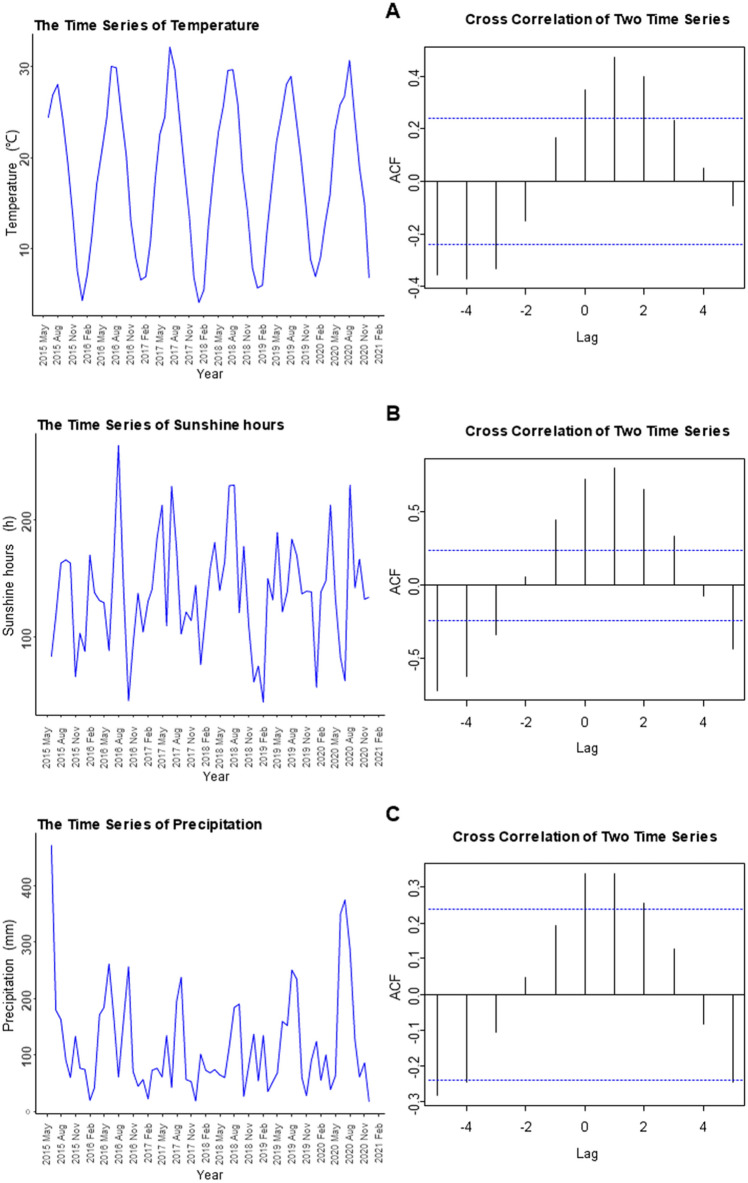
The correlation of meteorological factors with median serum 25(OH)D concentrations.

We used cross-correlation analysis to analyze the correlation between monthly median 25(OH)D values and monthly meteorological factors. Figure 3 shows the relationship between monthly meteorological data (including monthly average temperature, monthly average sunshine hours, and monthly total precipitation) and 25(OH)D levels. In the relationship between temperature and vitamin D, when the lag is equal to 1, the autocorrelation function (ACF) reached a maximum of 0.48, indicating a positive correlation between the temperature in the previous month and 25(OH)D. Similar cross-correlations were found for the mean monthly sunshine hours as well as the monthly total precipitation. No correlation was observed between other meteorological factors and 25(OH)D levels.

In addition, we performed univariate and multivariate logistic regression analyses. (We recalculated the average temperature and average sunshine hours for the 30 days before the pregnant women were examined as the meteorological indicator for the previous month.) As shown in Table 2, we found that mean temperature in the previous month may be an influencing factor for vitamin D deficiency. (OR = 0.92, *P* < 0.001), while no relationship was observed between monthly total precipitation, mean sunshine hours in the previous month and vitamin D deficiency. In the remaining parameters, vitamin D deficiency was significantly more probable in winter than in spring (OR = 1.47, *P* < 0.001). Age may be a risk factor for vitamin D deficiency (OR = 0.98, *P* < 0.001).

**Table 2:** Multivariate logistic regression analysis for associations with vitamin D deficiency.

Variables	Univariate odds ratio	95% CI		*P* value	Multivariate odds ratio	95% CI		*P* value
		Low	High			Low	High	
Age	0.98	0.97	0.99	< 0.001*	0.98	0.97	0.98	< 0.001*
Season (vs spring)								
Summer	0.37	0.34	0.41	< 0.001*	0.99	0.87	1.11	0.83
Autumn	0.29	0.26	0.31	< 0.001*	0.96	0.83	1.10	0.54
Winter	1.13	1.03	1.24	0.0091*	1.47	1.32	1.62	< 0.001*
Average temperature in previous month	0.92	0.91	0.92	< 0.001*	0.92	0.92	0.93	< 0.001*
Average sunshine hours in previous month	0.76	0.74	0.77	< 0.001*	0.98	0.96	1.01	0.25
Monthly total precipitation	0.90	0.89	0.91	< 0.001*	1.01	0.10	1.02	0.17

### Subgroup analysis of the association between meteorological factors and 25(OH)D levels

In Table 3, we could find that the relationship between mean temperature in the previous month and vitamin D deficiency persisted in different seasonal subgroups. (OR < 1 in all season subgroups) The relationship between age and vitamin D deficiency was also consistently observed. (OR < 1 in all season subgroups).

**Table 3:** Multivariate logistic regression analysis for associations with vitamin D deficiency in different season subgroups.

Subgroup(season)	Variables	Univariate odds ratio	95% CI		*P* value	Multivariate odds ratio	95% CI		*P* value
			Low	High			Low	High	
Spring (n = 5588)	Age	0.98	0.97	0.99	0.006*	0.98	0.97	1.00	0.01*
	Average temperature in previous month	0.97	0.96	0.98	< 0.001*	0.91	0.89	0.93	< 0.001*
	Average sunshine hours in previous month	1.02	0.97	1.08	0.43	1.36	1.25	1.49	< 0.001*
	Monthly total precipitation	1.06	1.01	1.11	0.03 *	1.17	1.10	1.23	< 0.001*
Summer (n = 5905)	Age	0.98	0.97	0.99	< 0.001*	0.98	0.96	0.99	< 0.001*
	Average temperature in previous month	0.91	0.90	0.92	< 0.001*	0.91	0.90	0.92	< 0.001*
	Average sunshine hours in previous month	0.91	0.88	0.95	< 0.001*	0.98	0.94	1.03	0.40
	Monthly total precipitation	1.01	1.00	1.03	0.10	1.03	1.01	1.04	0.008*
Autumn (n = 5435)	Age	0.97	0.96	0.99	< 0.001*	0.97	0.96	0.99	< 0.001*
	Average temperature in previous month	0.86	0.84	0.88	< 0.001*	0.84	0.81	0.86	< 0.001*
	Average sunshine hours in previous month	0.85	0.82	0.87	< 0.001*	1.07	1.01	1.13	0.02* ara>
	Monthly total precipitation	0.96	0.94	0.97	< 0.001*	1.01	0.98	1.03	0.61
Winter (n = 5162)	Age	0.97	0.96	0.99	< 0.001*	0.98	0.97	1.00	0.02*
	Average temperature in previous month	0.93	0.92	0.94	< 0.001*	0.95	0.93	0.96	< 0.001*
	Average sunshine hours in previous month	0.69	0.64	0.73	< 0.001*	0.85	0.78	0.92	< 0.001*
	Monthly total precipitation	1.04	0.99	1.10	0.14	1.04	0.98	1.10	0.20

We divided the age into three subgroups (< 25 years old, 25–35 years old, and > 35 years old) to observe the association between meteorological factors and vitamin D deficiency in pregnant women of different ages. We consistently observed the relationship between the mean temperature in the previous month and vitamin D deficiency in Table 4. (OR < 1 in all age subgroups).

**Table 4:** Multivariate logistic regression analysis for associations with vitamin D deficiency in different age subgroups.

Subgroup (age)	Variables	Univariate odds ratio	95% CI		*P* value	Multivariate odds ratio	95% CI		*P* value
			Low	High			Low	High	
< 25 (n = 2319)	Season (vs spring)								
	Summer	0.28	0.19	0.42	< 0.001*	0.98	0.53	1.82	0.95
	Autumn	0.20	0.13	0.30	< 0.001*	0.96	0.48	1.93	0.92
	Winter	1.05	0.63	1.75	0.86	1.58	0.90	2.78	0.11
	Average temperature in previous month	0.89	0.88	0.91	< 0.001*	0.90	0.86	0.93	< 0.001*
	Average sunshine hours in previous month	0.77	0.70	0.86	< 0.001*	1.06	0.93	1.20	0.38
	Monthly total precipitation	0.89	0.85	0.93	< 0.001*	1.02	0.96	1.08	0.55
25–35(n = 16,531)	Season (vs spring)								
	Summer	0.39	0.35	0.43	< 0.001*	0.99	0.85	1.15	0.85
	Autumn	0.30	0.27	0.33	< 0.001*	0.96	0.81	1.14	0.63
	Winter	1.16	1.03	1.30	0.02 *	1.48	1.27	1.63	< 0.001*
	Average temperature in previous month	0.92	0.92	0.92	< 0.001*	0.93	0.92	0.94	< 0.001*
	Average sunshine hours in previous month	0.77	0.75	0.79	< 0.001*	1.00	0.96	1.03	0.83
	Monthly total precipitation	0.90	0.89	0.91	< 0.001*	1.01	0.99	1.02	0.36
> 35(2340)	Season (vs spring)								
	Summer	0.36	0.31	0.42	< 0.001*	1.00	0.81	1.24	0.98
	Autumn	0.27	0.23	0.31	< 0.001*	0.97	0.76	1.23	0.78
	Winter	1.11	0.95	1.30	0.20	1.53	1.28	1.83	< 0.001*
	Average temperature in previous month	0.91	0.91	0.92	< 0.001*	0.92	0.91	0.94	< 0.001*
	Average sunshine hours in previous month	0.73	0.70	0.76	< 0.001*	0.95	0.91	1.00	0.04*
	Monthly total precipitation	0.90	0.89	0.92	< 0.001*	1.01	0.99	1.03	0.32

### Non-linear relationship between mean temperature in the previous month and 25(OH)D levels

**Figure 4:**
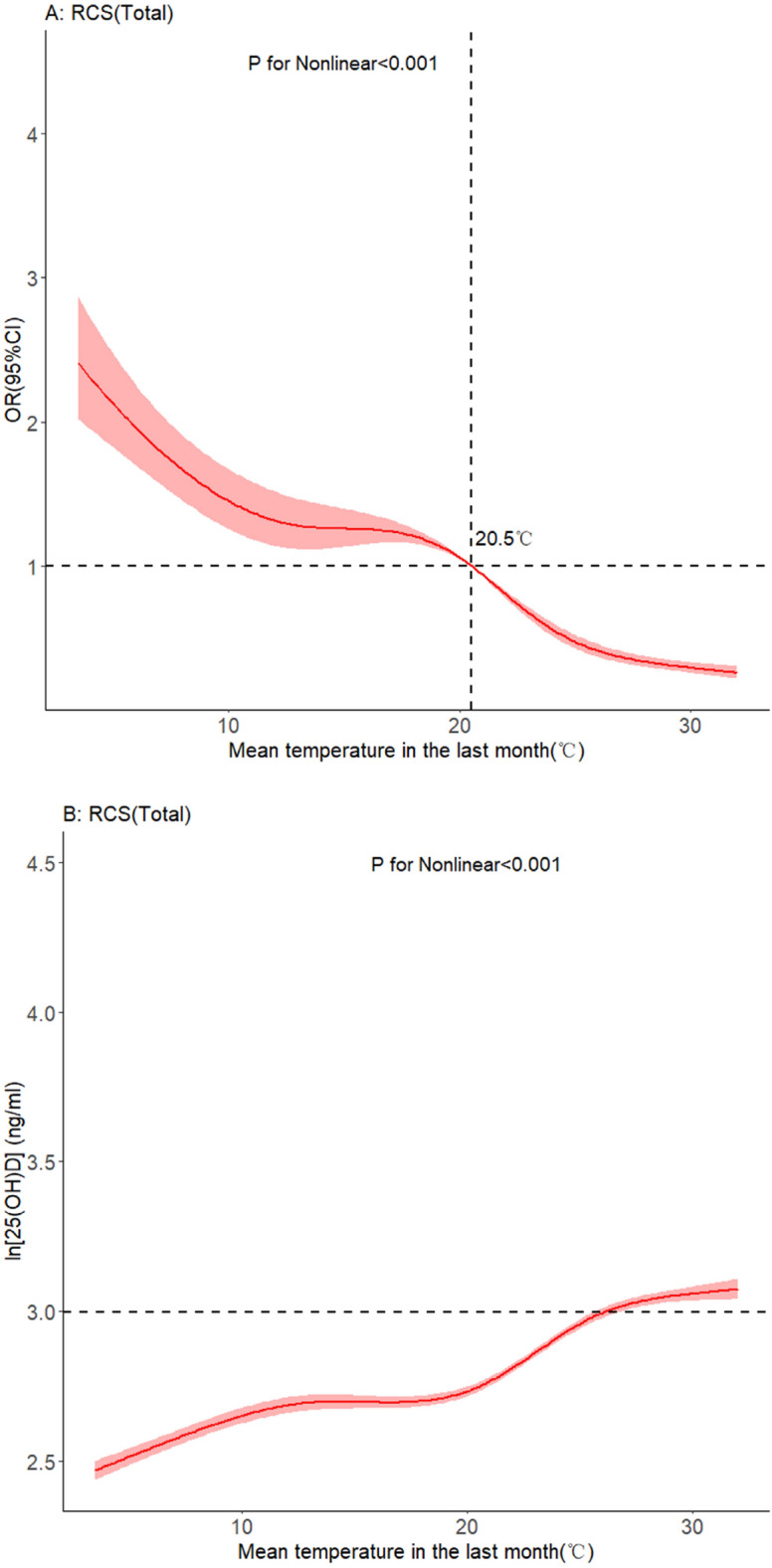
Dose–response relationship between mean temperature and serum 25(OH)D levels.

In fitting the non-linear relationship, we first took the natural logarithm of 25(OH)D to ensure the normality of the data (a plot of the probability density distribution before and after log transformation can be seen in Figure S1). A multivariate-adjusted spline regression model established the non-linear relationship as Fig. 4A shows. (In the model, we adjusted for age, season, and sunshine hours) Fig. 4B shows the non-linear relationship between average temperature in the previous month and log 25(OH)D level.

**Figure 5:**
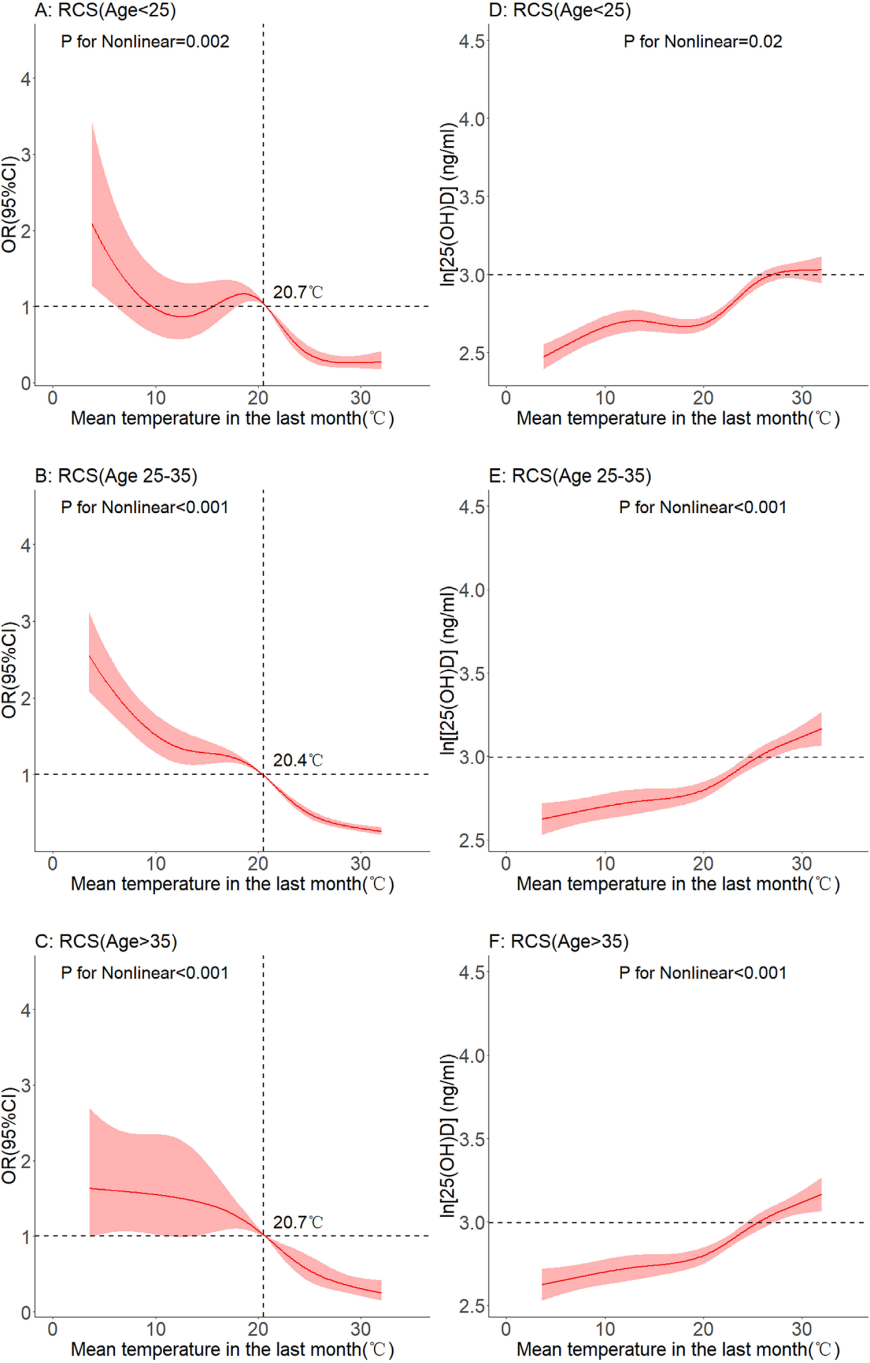
Dose–response relationship between mean temperature and serum 25(OH)D levels. (Age subgroup).

In subgroup analyses grouped by different ages (< 25 years old, 25–35 years old, > 35 years old), a non-linear relationship was observed between average temperature in the previous month and vitamin D deficiency. As shown in Fig. 5A,C and E, as the temperature increased to 20 °C, the OR value became less than 1. As shown in Fig. 5B,D and F, 25(OH)D levels increased with increasing temperature.

**Figure 6:**
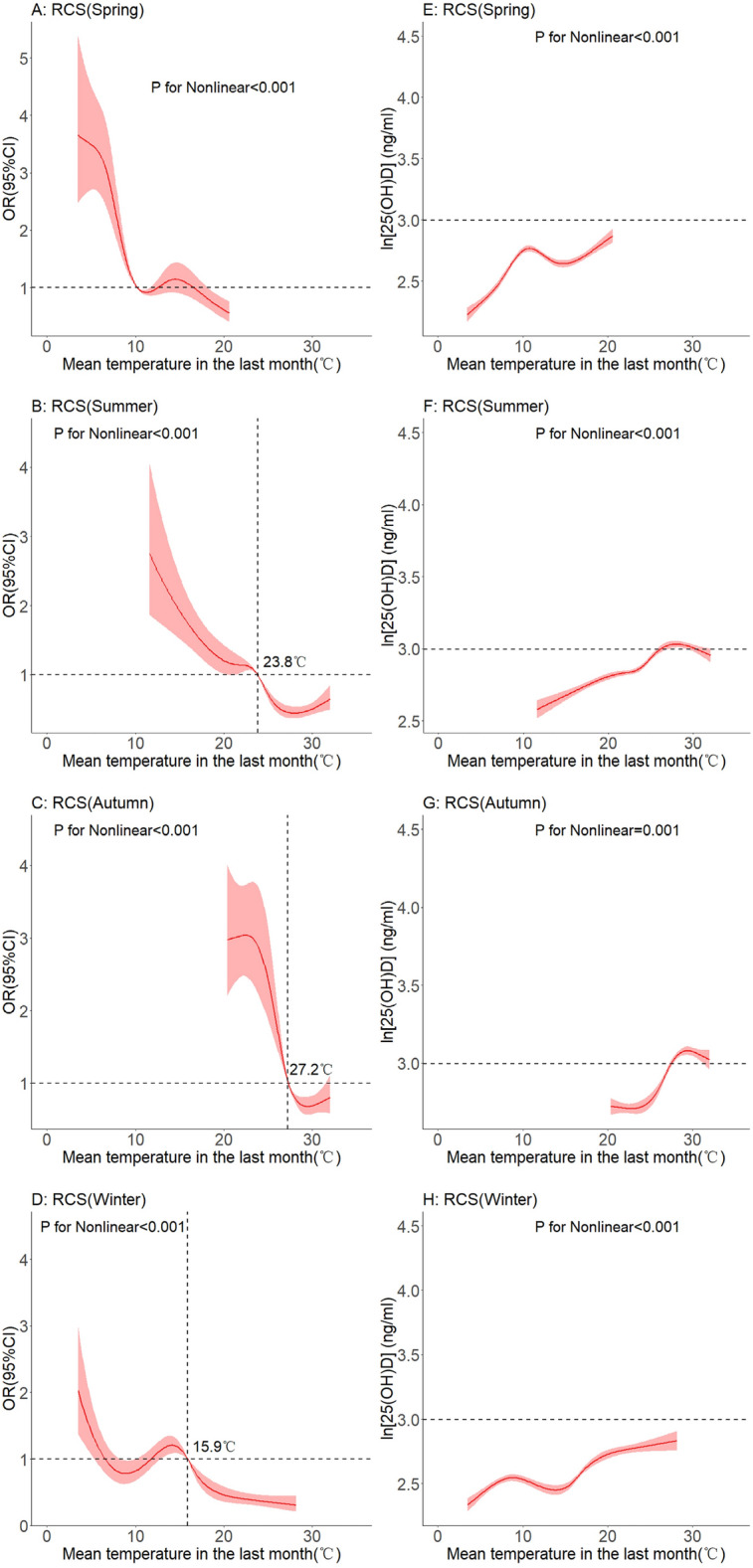
Dose–response relationship between mean temperature and serum 25(OH)D levels. (Seasonal subgroup).

In a subgroup analysis of the different seasons, a non-linear relationship was found between average temperature in the previous month and vitamin D deficiency. As shown in Fig. 6A,C,E G, with the increase of temperature, the OR value gradually becomes less than 1. As shown in Fig. 6B,D,F, and H, there was a non-linear relationship between average temperature in the previous month and log 25(OH)D levels in different seasons. In general, the level of 25(OH)D increased with increasing temperature. However, this upward trend became flat or unobservable after rising to a certain temperature in spring and summer. A downward trend was observed in the autumn.

### Relationship between mean temperature of the same month and 25(OH)D levels

We have additional research on the temperature of the same month in the supplement. We found that mean temperature of the same month is a protective factor for vitamin D deficiency. (OR = 0.92(not adjusted), OR = 0.93(adjusted for age, season, sunshine hours and precipitation)) Neither the non-linear relationship between temperature of the same month and vitamin D deficiency nor the non-linear relationship between temperature and vitamin D levels was observed. In the subgroup analyses of summer and autumn, we also observed a potential inverted U-shaped relationship between temperature and vitamin D levels in the same month.

## Discussion

Demographic information on the vitamin D levels of pregnant women in eastern China is limited. From 2015 to 2020, 65.68% of pregnant women in the Kunshan area had 25(OH)D levels above 50 nmol/l. Despite being located in a region of eastern China that receives adequate sunshine throughout the year, a considerable proportion of women in this region still have low vitamin D. As the economy and medical care have developed, the general health of pregnant women has improved, including their vitamin status. Overall 25(OH)D status in pregnant women increased from 2015 to 2018, but this upward trend has leveled off in recent years. One possible reason for the trend of slow growth in 25(OH)D status is the extreme hot weather in recent years. In extremely hot weather, we speculate that the outside habits of pregnant women may change, potentially affecting the amount of UV radiation they receive. This change in going outside habits may affect the vitamin D status.

By analyzing the relationship between meteorological factors and 25(OH)D levels, we found that temperature was an important influencing for vitamin D deficiency. The relationship between temperature and 25(OH)D levels persisted in different subgroups. Based on such findings, we further explored the non-linear relationship between mean temperature in the previous month and vitamin D deficiency (and log 25(OH)D). The change of 25(OH)D concentration with increasing temperature was small until the temperature reached 20℃. While between 20 °C and 25 °C, this variation is wide. It was probable that the pregnant women had more outdoor activities in this comparatively more comfortable temperature range. Then when the temperature exceeded 25 °C, pregnant women spent progressively less time outdoors, which may have contributed to the lower 25(OH)D change. The different definition of vitamin D deficiency had little effect on the non-linear relationship between temperature and vitamin D. However, different definitions of vitamin D can lead to huge variations in the OR of temperature. A low criterion will overestimate the influence of temperature (the OR will be particularly high) and a high criterion will underestimate the influence of temperature (the OR will be particularly close to 1).

Our study provides further population-based information on vitamin D levels in pregnant women in southeastern China. Despite being located in sunny eastern China, the vitamin D levels of pregnant women were generally inadequate. A previous study assessed the status of overall vitamin D levels in pregnant women in China based on data from the China Nutrition and Health Surveillance (CHNS), with median 25(OH)D concentrations of 15.48 (11.89–20.09) ng/mL in 2010–2012 and 2015 –2017 was 13.02 (10.17–17.01) ng/mL, Vitamin D adequacy was only 25.17% in 2010–2011 and 12.57% in 2015–2017^37^. Although, we took different serum 25(OH)D thresholds to define vitamin D deficiency and insufficiency. We still observed chronic vitamin D deficiency and insufficiency in Chinese pregnant women. Studies from India^38^ and Bangladesh^39^ have also reported a very high prevalence of vitamin D deficiency and insufficiency in pregnant women. Vitamin D deficiency in pregnant women continues to be a severe public health problem in tropical and subtropical regions.

In addition, in a previous study, researchers found that maternal vitamin D deficiency was positively associated with Hui ethnicity (*P* = 0.02, relative to Han ethnicity), vitamin D supplementation (*P* = 0.02) and low ambient UV levels (*P* < 0.001). As most of the serum 25(OH)D samples were taken in autumn and winter, seasonal factors were not included in the logistic regression. Still, additional analyses were conducted for autumn and winter samples^40^. A study from Shenzhen, China, showed that season was also an associated factor for vitamin D deficiency in pregnant women. (Winter vs autumn vs spring, OR = 3.69, *P* < 0.001)^41^. Other studies report the association between BMI^42^, gestational frequency^43,44^ and gestational age^39,45–47^ and maternal vitamin D deficiency. However, to our knowledge, few studies have focused on exploring the relationship between meteorological factors and vitamin D under different seasons.

The current study's novelty is that the relationship between average temperature in the previous month and 25(OH)D levels varies across the seasons. Specifically, the upward trend in 25(OH)D levels became less pronounced in the spring and summer after reaching certain temperatures. In particular, in autumn, when the average temperature reached 29 °C in the previous month, 25(OH)D levels started to decrease. In recent years, in southeastern China, maximum temperatures reached around 40 °C in summer and autumn. We speculate that excessively high temperatures may lead to fewer pregnant women going outside. They may therefore lack the necessary amount of sunshine and exercise, which may contribute to lower vitamin D levels^48^. In addition, external vitamin supplementation is an essential factor affecting the vitamin D levels in the human body. And temperature may affect the content of vitamin D supplements. Due to environmental factors such as temperature^49^, oxygen and light, vitamin D may be lost during food processing and storage^50^.

Vitamin D levels in pregnant women are critical to their clinical outcomes. Our study assessed the relationship between meteorological factors and 25(OH)D levels through a retrospective study of a large population. Our study took complete account of the seasonality of 25(OH)D levels in the people. However, some limitations of our analysis remain. Although our study included healthy women of the same gestational age, other information, such as BMI and whether sun protection, was not collected. Secondly, the clues obtained in this paper can only indicate the correlation between factors and vitamin D. The genuine causal relationship needs to be verified through further studies. Thirdly, the measurement of serum 25(OH)D concentrations is not the golden standard, which may have an influence on the analysis results.

In conclusion, our study found that vitamin D levels in pregnant women in southeast China remain inadequate. Temperature in the previous month is a factor associated with 25(OH)D levels, but it behaves differently under different seasons. In spring, summer and autumn, temperature and vitamin D levels in pregnant women showed a potential inverted U-shaped relationship: too high or too low temperatures led to lower 25(OH)D levels. Our findings may have important implications for public health strategies involving pregnant women in China.

## Supplementary Information

Supplementary Information.

## Data Availability

The original contributions presented in the study are included in the article. Further inquiries can be directed to the corresponding authors.
