# Supplementary material for: Correlation between meteorological factors and vitamin D status under different season
Source: Sci Rep. 2023 Mar 23;13:4762. doi: 10.1038/s41598-023-31698-2 (PMC10036626; doi:10.1038/s41598-023-31698-2)

**Table S1.** Multivariate logistic regression analysis for associations with vitamin D deficiency

| Variables | Univariate Odds ratio | 95% CI | | P value | Multivariate Odds ratio | 95% CI | | P value |
| --- | --- | --- | --- | --- | --- | --- | --- | --- |
|  |  | Low | High |  |  | Low | High |  |
| Age | 0.98 | 0.97 | 0.99 | <0.001^*^ | 0.98 | 0.97 | 0.98 | <0.001^*^ |
| Season (vs spring) |  |  |  |  |  |  |  |  |
| Summer | 0.37 | 0.34 | 0.41 | <0.001^*^ | 0.91 | 0.81 | 1.02 | 0.12 |
| Autumn | 0.29 | 0.26 | 0.31 | <0.001^*^ | 0.66 | 0.59 | 0.75 | <0.001^*^ |
| Winter | 1.13 | 1.03 | 1.24 | 0.01^*^ | 0.99 | 0.89 | 1.09 | 0.78 |
| Average temperature | 0.92 | 0.91 | 0.92 | <0.001^*^ | 0.93 | 0.92 | 0.94 | <0.001^*^ |
| Sunshine hours | 0.81 | 0.79 | 0.82 | <0.001^*^ | 0.97 | 0.94 | 1.00 | 0.07 |
| Average precipitation | 0.90 | 0.89 | 0.91 | <0.001^*^ | 1.00 | 0.98 | 1.01 | 0.91 |

**Figure S1:** Probability density plot of 25(OH)D data.


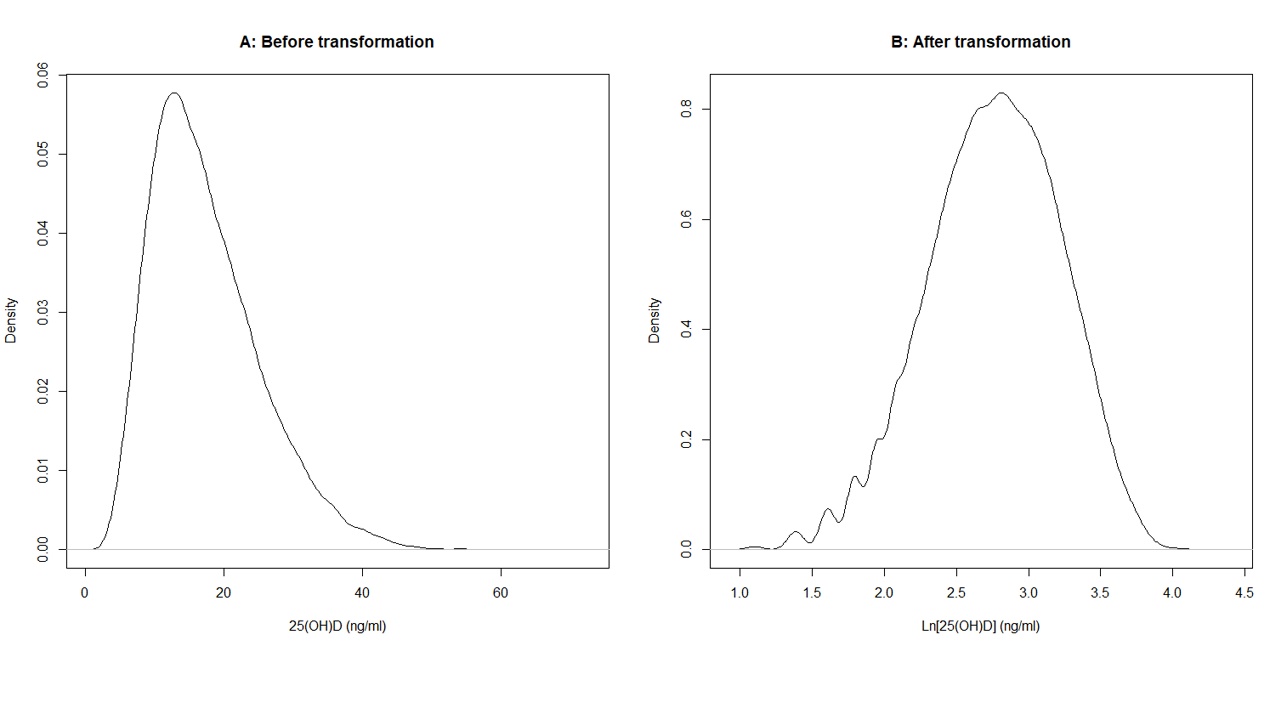


**Figure S2:** Dose-response relationship between mean temperature of the same month and serum 25(OH)D levels.

**
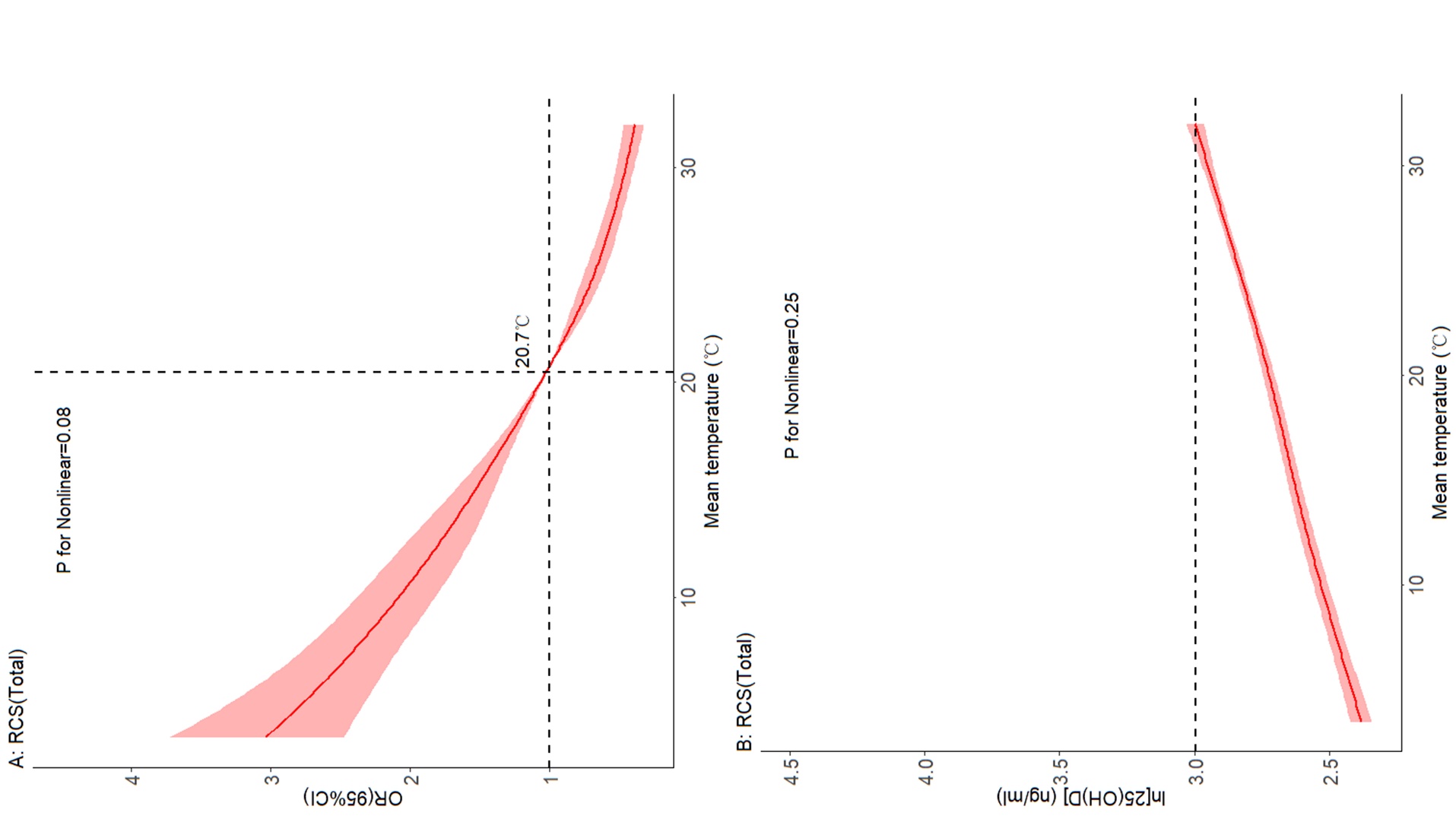
**

**Figure S3:** Dose-response relationship between mean temperature of the same month and serum 25(OH)D levels. (Age subgroup)


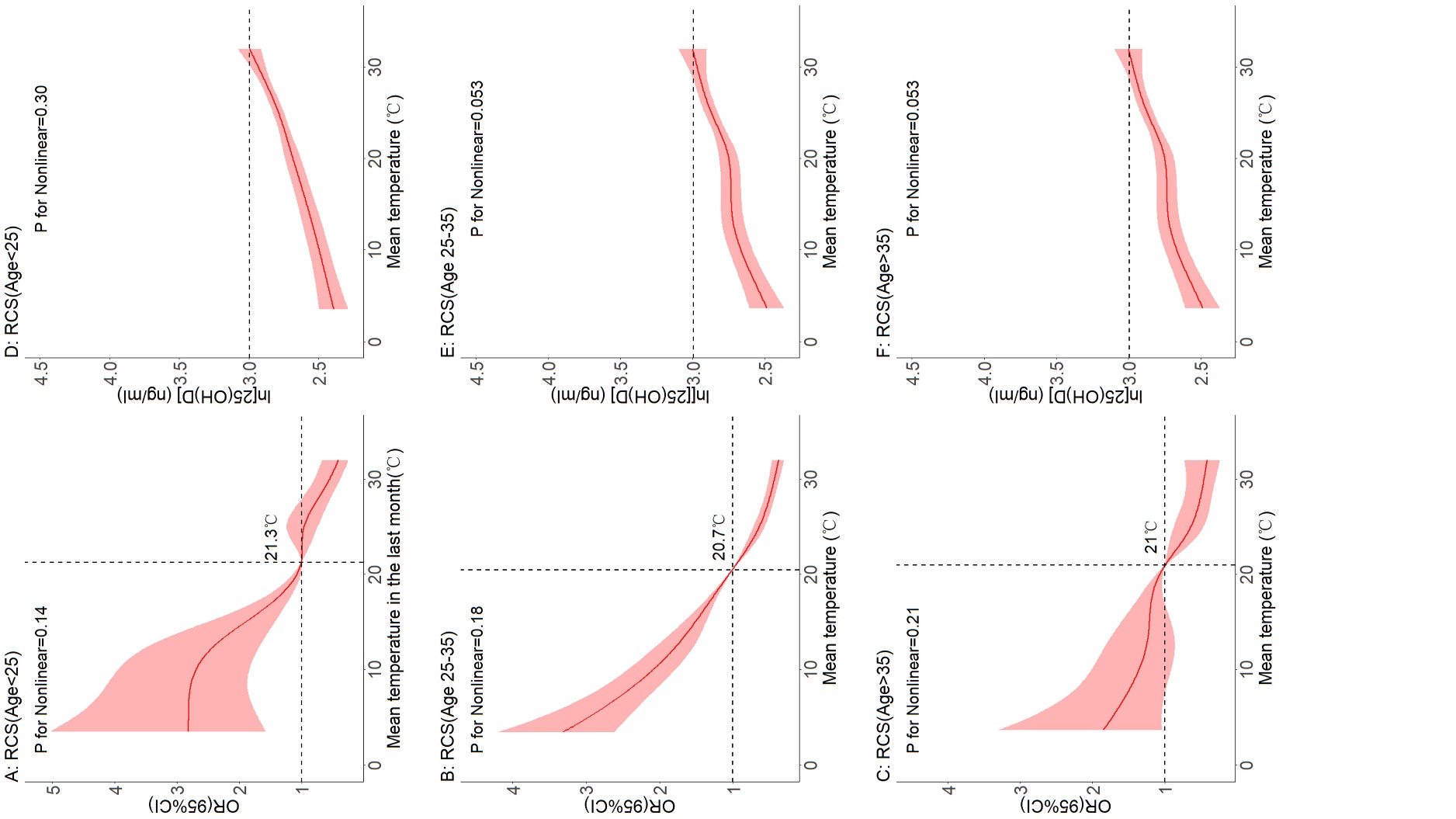


**Figure S4:** Dose-response relationship between mean temperature of the same month and serum 25(OH)D levels. (Seasonal subgroup)


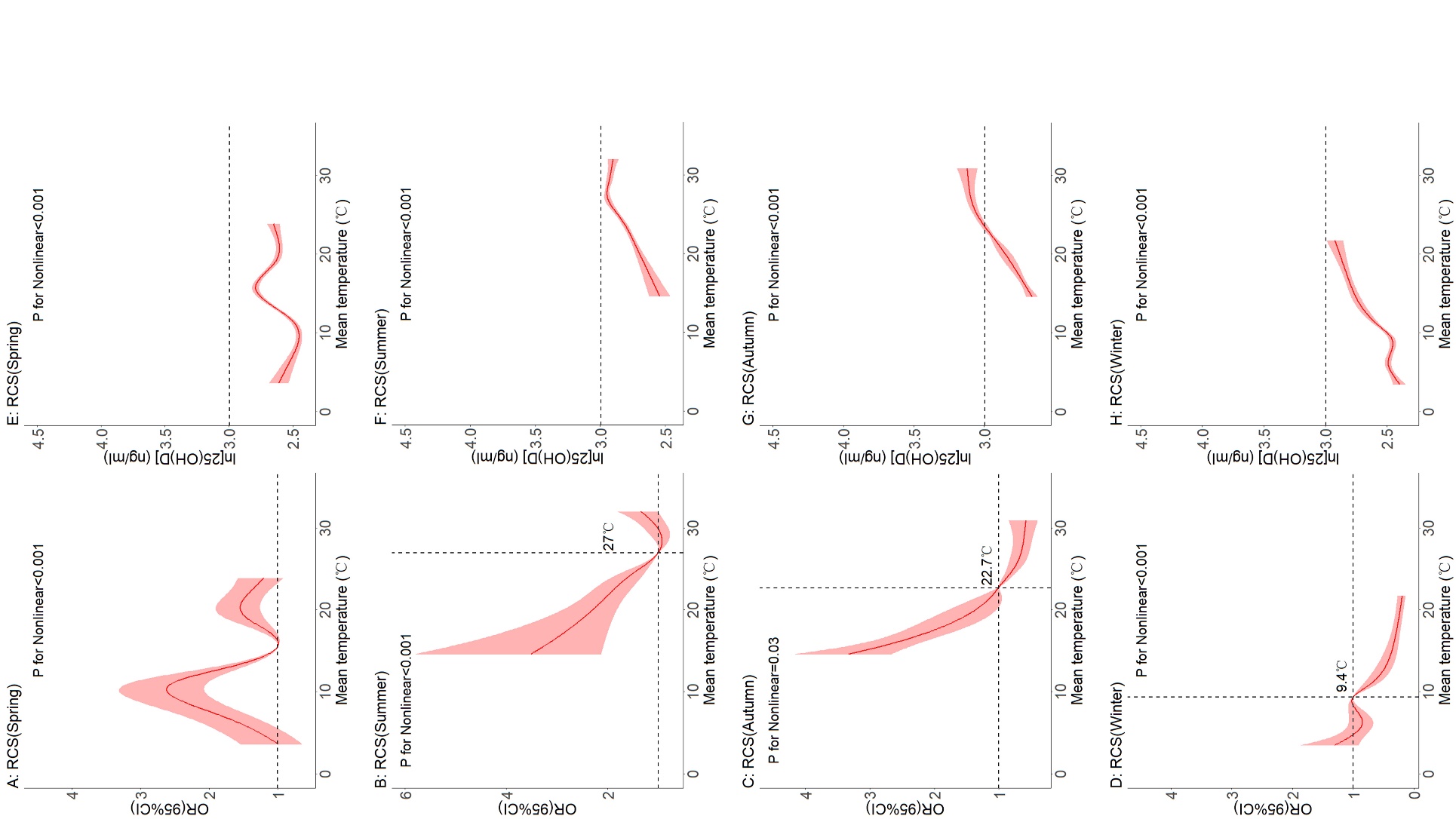

Supplement: Supplementary file 1 — Supplementary Information. [file 41598_2023_31698_MOESM1_ESM.docx]
